# Systematic analysis of histone acetylation regulators across human cancers

**DOI:** 10.1186/s12885-023-11220-7

**Published:** 2023-08-08

**Authors:** Congkuan Song, Xinfei Liu, Weichen Lin, Kai Lai, Shize Pan, Zilong Lu, Donghang Li, Ning Li, Qing Geng

**Affiliations:** 1 Department of Thoracic Surgery, Renmin Hospital of Wuhan University, No.238 Jiefang Road, Wuchang District, Wuhan, 430060 China; 2 Department of Hematology, Renmin Hospital of Wuhan University, No.238 Jiefang Road, Wuchang District, Wuhan, 430060 China

**Keywords:** Histone acetylation (HA) regulators, Cancer, Genetic alterations, Hallmark pathways, Survival, HDAC3

## Abstract

**Background:**

Histone acetylation (HA) is an important and common epigenetic pathway, which could be hijacked by tumor cells during carcinogenesis and cancer progression. However, the important role of HA across human cancers remains elusive.

**Methods:**

In this study, we performed a comprehensive analysis at multiple levels, aiming to systematically describe the molecular characteristics and clinical relevance of HA regulators in more than 10000 tumor samples representing 33 cancer types.

**Results:**

We found a highly heterogeneous genetic alteration landscape of HA regulators across different human cancer types. CNV alteration may be one of the major mechanisms leading to the expression perturbations in HA regulators. Furthermore, expression perturbations of HA regulators correlated with the activity of multiple hallmark oncogenic pathways. HA regulators were found to be potentially useful for the prognostic stratification of kidney renal clear cell carcinoma (KIRC). Additionally, we identified HDAC3 as a potential oncogene in lung adenocarcinoma (LUAD).

**Conclusion:**

Overall, our results highlights the importance of HA regulators in cancer development, which may contribute to the development of clinical strategies for cancer treatment.

**Supplementary Information:**

The online version contains supplementary material available at 10.1186/s12885-023-11220-7.

## Introduction

Cancer is a major public health problem in the whole world, with high global morbidity and mortality, causing enormous economic burden and distracting human development. Beyond genome alternations were initially thought as the driver of carcinogenesis, cancer mechanisms have expended to a complex roster including genomic and non-genomic derangements [1]. Epigenetic modifications are functioned as heritable and reversible alternations in genes or genomes without any changes in primitive DNA sequence, and thus act as a vital role in modulation of gene transcription. Emerging evidence highlights that epigenomic dynamics are implicated in regulation of cancers initiation, progression, metastasis and even impacting status of immune cells in the context of cancer immunotherapy[2]. Regard this as cutting point, exploration of epigenetic adaptation in cancer transformation and epidrugs discovery is a burgeoning field.

Epigenetic regulation, including DNA and histone modifications, non-coding RNA modulation and chromatin remodeling, exerts epigenomic imprinting on genomic DNA and endows general flexibility and plasticity on gene expression. As the decisive advances in decrypting mechanistic of cancerization processes, the cellular memory of epigenetic modifications is lost or perturbated in the circumstances of tumor occurrence and development. Histone acetylation (HA), an important and common epigenetic pathway, regulates gene transcription at relevant genomic sites by controlling the tension of nucleosome structure. Thus, there is no doubt that this epigenetic mechanism could be hijacked by tumor cells during carcinogenesis and cancer progression. As the actual fact is, growing evidence shows that the perturbations on histone acetylation are commonly observed in the cases of tumor occurrence [3–5].The “eraser” of HA HDAC1 has been reported to be highly expressed in non-small cell lung cancer (NSCLC) cell lines, and down-regulation of HDAC1 inhibits lung cancer cell proliferation, migration, invasion, tumor angiogenesis, and induces cell apoptosis [6]. And the up-regulation of HDAC2 in breast cancer cells and tissues can also regulate the malignant biological behavior of breast cancer cells. In addition, HA dynamics also regulates immune-related processes under tumor microenvironment and could be harnessed stochastically for evasion of immunosurveillance [7]. In addition, HA dynamics also regulates immune-related processes under tumor microenvironment and could be harnessed stochastically for evasion of immunosurveillance [8]. These findings suggest that HA regulators are involved in important biological processes in cancer.

Studies on histone modifications have never stopped. However, the important role of HA across human cancers remains elusive. Decrypting the landscape and gaining insights of the underlying mechanisms of HA could help delineate alternative and preventive strategies for clinical cancer therapy. Thus, in this study, we performed a comprehensive analysis at multiple levels, aiming to systematically describe the molecular characteristics and clinical relevance of HA regulators in 33 cancer types. We found that there were extensive genetic alterations in HA regulators in human cancers. We also evaluated the correlation of expression perturbations of HA regulators with the activity of cancer pathways, and explored the clinical prognostic value of HA regulators. Overall, our comprehensive analysis of HA regulators provides important resources for understanding HA biology.

## Materials and methods

### Data source and preprocessing

Histone acetylation (HA) regulators (Table S1) were identified from a previous report [9]. The FPKM-based mRNA expression data, TCGA thresholded SCNA scores (ISAR_GISTIC.all_data_by_genes.txt), somatic mutation data, and clinical data were download from the Genomic Data Commons (https://portal.gdc.cancer.gov/), Broad GDAC Firehose (https://gdac.broadinstitute.org/) and the Xena Browser (https://xenabrowser.net/datapages/). The GDC.h38 GENCODE v22 GTF (gencode.v22.annotation.gtf.gz) (https://gdc.cancer.gov/download-gdc-reference-files) was used as the genome annotation file.

### Differential expression analysis and Pearson correlation analysis

From 33 cancer types, 19 cancer types with at least 3 matched tumors and normal samples were selected for differential expression analysis. Differentially expressed genes (DEGs) were defined as described by Li et al.[10]. The Wilcox’s test was used to identify DEGs with adjusted p-values by BH method. The threshold was set as the adjusted p-value less than 0.05. The Pearson correlation between somatic copy number alterations and the expression of HA regulators were investigated as described by Liu et al.[11]. The correlation with p-value less than 0.05 was considered as significant or considerable.

### Hallmark pathway activity across cancer types

Gene Set Variation Analysis (GSVA) is a non-parametric unsupervised analysis method, which is mainly used to evaluate whether different metabolic pathways are enriched between different samples by converting the expression matrix of genes between different samples into the expression matrix of gene sets between samples. Unlike gene-set enrichment analysis (GSEA), GSVA does not require pre-grouping of samples and can calculate enrichment scores for specific gene sets in each sample. Here, GSVA was performed on the normalized gene expression to calculate the hallmark pathway activity with GSVA scores. To identify the HA regulators associated with pathway activation or inhibition, we calculated Pearson correlation coefficient (PCC) between the HA regulators expression and pathway activity. The HA regulators with significant correlations were defined as those with absolute value of regulator-pathway pair correlation coefficients greater than 0.2 and adjusted p-value less than 0.001.

### Cross talks among histone acetylation (HA) regulators

Based on the gene expression of 33 cancer types, we calculated the PCC among HA regulators and visualized them with “corrplot” R package. Furthermore, we also investigated the protein-protein interaction (PPI) networks among these HA regulators through the String database (https://cn.string-db.org/) (Browsing date: October 7, 2022). This interaction was subsequently further visualized in the Cytoscape_v3.9.0. software.

### Survival analysis and CellMiner analysis for HA regulators

To investigate the correlation between the expression of HA regulators and patient survival, we divided all patients into two groups according to the median expression of each HA regulator. Cox regression was used to examine the differences in survival between the two groups. In this process, the “survival” package in R was adopted. In addition, in order to screen potential therapeutic agents targeting HA regulators, we downloaded drug information and sequencing data from the CellMiner database (https://discover.nci.nih.gov/cellminer/home.do). Here, we calculated the PCC between the expression level of each HA regulator and drug half inhibitory concentration (IC50).

### Unsupervised consensus clustering

Based on the mRNA expression of HA regulators in KIRC tumor samples, we performed unsupervised consensus clustering to identify different patient clusters. The “ConsensuClusterPlus” package in R was adopted. In this process, 1000-time repetitions were performed to guarantee the classification stability. The specific setting parameters were shown as follows: maxK = 5, reps = 1000, pItem = 0.8, pFeature = 1, seed = 73, clusterAlg = “km”, distance = “euclidean”.

### Cell culture

A549, H1299 and Beas-2B cells were all derived from the ATCC. A549 cells were cultured in F-12k medium, H1299 in RPMI 1640 medium, and human normal bronchial epithelial cells Beas-2B in DMEM medium. In these media, we supplemented them with 10% fetal bovine serum (FBS) and 1% streptomycin and 1% penicillin. The cells were cultured in a cell incubator at 37℃ and 5%CO_2_.

### Cell transfection

The cell density of 2.5 × 10^5^ cells per well was inoculated in 6-well plate, and siRNA transfection was performed after A549 cells were attached to the wall at 70–90% confluent. Transfection experiment was divided into four groups: negative control group (si-NC), si-HDAC3-001, si-HDAC3-002 and si-HDAC3-003. The si-HDAC3-001 primer sequence was 5’-GAGCAACCCAGCTGAACAA-3’, si-HDAC3-002 primer sequence was 5’-GTCCTGCATTACGGTCTCT-3’, and si-HDAC3-003 primer sequence was 5’-GTGGTTATACTGTCCGAAA-3’. The transfection procedure was carried out according to LipofectamineTM2000 instructions.

### Real time quantitative PCR (RT-qPCR)

Total RNA extraction of cells was performed by TRIpure Total RNA Extraction Reagent (Biosharp, China). RNA was subsequently reverse transcribed to cDNA according to the cDNA Synthesis Kit (Servicebio, Wuhan, China). HDAC3 primer sequence: S: 5’-TACGGAGCTGGACACCCTATG-3’, A: 5’-ATGTAGTCCTCGGAGTGGAAGC-3’. GAPDH primer sequence: S: 5’-GGAAGCTTGTCATCAATGGAAATC-3’, A: 5’-TGATGACCCTTTTGGCTCCC-3’. RT-qPCR was performed using validated specific primers and SYBR Green PCR SuperMix (Servicebio, China). Meanwhile, GAPDH was used as an internal reference to calculate the mRNA content of target gene by 2^−ΔΔCt^ method.

### Western blots

Cells were treated with RIPA cell lysis buffer containing PMSF. The protein concentration was determined by BCA method, and the protein solution and 5×Loading Buffer were subsequently mixed according to the volume ratio of 4∶1, and boiled at 100 ℃ for 10 min. After SDS-PAGE gel, 50 µg of total protein samples were added to each sample well for electrophoresis for 1.5 h and a voltage of 100 V. The membrane was transferred at 4 ℃ and 250 mA for 2 h. NC membranes were immersed in 5% skim milk for 1.5 h at room temperature. The NC membranes were then placed within the diluted primary antibody at 4℃ overnight. After this, the NC membranes were placed within the diluted secondary antibody (1:10000) for 2 h. HDAC3, E-cadherin, Vimentin, N-cadherin and GAPDH were diluted at 1: 600, 1 : 1000, 1: 1000, 1: 1000 and 1: 1000 respectively. ECL chemiluminescence solution was used for protein exposure.

### CCK-8 assay

A549 cells in the logarithmic growth phase were digested with trypsin (Servicebio, Wuhan, China). Then cells were collected, centrifuged and resuspended with F-12k culture medium. The cell suspension were seeded in 96-well plates at 8000 cells/well, with each group repeated three wells and cultured for 24 h. The RNA transfection was carried out according to LipofectamineTM2000 instructions. Cells were cultured for 24 h, the CCK-8 solution was added at 10 µl/well, and the cells were subsequently incubated in an incubator for 2 h at 37℃ and 5%CO_2_. The cell proliferation rate was calculated by measuring the OD of each well at 450 nm.

### EDU assay

At 48 h after transfection of A549 cells, cell suspension was configured after trypsin digestion and seeded in 6-well plates at a density of 1*10^5^ cells/well and cultured overnight. They were supplemented with 10 µmol/L EdU for further 2 h. Cells were fixed with 4% paraformaldehyde for 30 min and permeabilized with 0.5%Triton X-100 permeabilized solution for 20 min at room temperature. Cells were stained with Apollo staining solution for 30 min and DAPI for 5 min. Photo was taken under a fluorescence microscope. DAPI positive cells (blue) are the total cell number and EdU positive cells (red) are the proliferating cells.

### Wound healing assay

Three repeated wells were set for the experimental and control groups. A549 cells from the logarithmic growth phase were seeded at 500 µL per well (2*10^5^/mL) in 6-well plates. Then each well was added with 2 mL of F-12k complete medium and cells were placed in an incubator at 37℃ and 5%CO_2_. After the cells covered the bottom of the plate, we used a 1 ml pipette tip to make scratches on the well plate. The scratch widths at 0 and 24 h were subsequently observed and photographed.

### Transwell assay

The BD matrigel and F-12k were diluted in a 1:3 ratio, then 50ul was absorbed into the transwell upper chamber and placed in an incubator for about 4 h. A549 cells in the experimental and control groups were seeded in a small Transwell chamber with 0.2 mL per well (2*10^5^/mL), with three repeated wells for each group. After adding 500 µl of complete medium to the Transwell plate, the chamber with A549 cells was put into the plate and cultured in a 37℃ incubator for 24 h. The medium in the chamber was then removed and rinsed with PBS and stained with crystal violet for 10 min. The redundant crystal violet on the surface of the chamber was cleaned with running water, and the cells in the upper chamber were wiped clean with cotton swabs. The non-cell inoculant side was photographed, and three fields were randomly selected under an inverted microscope (×100) to calculate the number of cells.

### Bioinformatic analysis of HDAC 3 expression differences in LUAD

Two LUAD chips (GSE10072 [12], and GSE32863 [13]) were downloaded from Gene Expression Omnibus (https://www.ncbi.nlm.nih.gov/geo/) to obtain gene expression data for HDAC3, and we divided all patients into two groups according to the median expression of HDAC3. Statistical differences between the two groups were calculated using Wilcoxon rank sum test. The data was visualized with the “ggplot2” R package [14]. GSE10072 dataset included transcription profile data of 49 normal lung tissues and 58 lung tumor tissues. And GSE32863 dataset included transcription profiling data from 58 normal lung tissues and 58 lung tumor tissues. The difference in protein expression of HDAC3 between LUAD tumors and normal tissues was explored using the UALCAN database (http://ualcan.path.uab.edu/analysis-prot.html). To further verify the differential expression of HDAC3 protein in LUAD, immunohistochemical sections from LUAD tumor and normal tissues were also obtained from the Human Protein Atlas (https://www.proteinatlas.org).

### Statistical analysis

Gene differential expression analysis was performed by the “limma” R package. The Wilcoxon rank-sum test was used to calculate the statistical differences between the two groups. Differences in survival between two or more groups was compared using the Kaplan-Meier method. A P-value of < 0.05 was considered statistically significant.

## Results

### Genetic alterations in HA regulators in human different cancer types

**Fig. 1:**
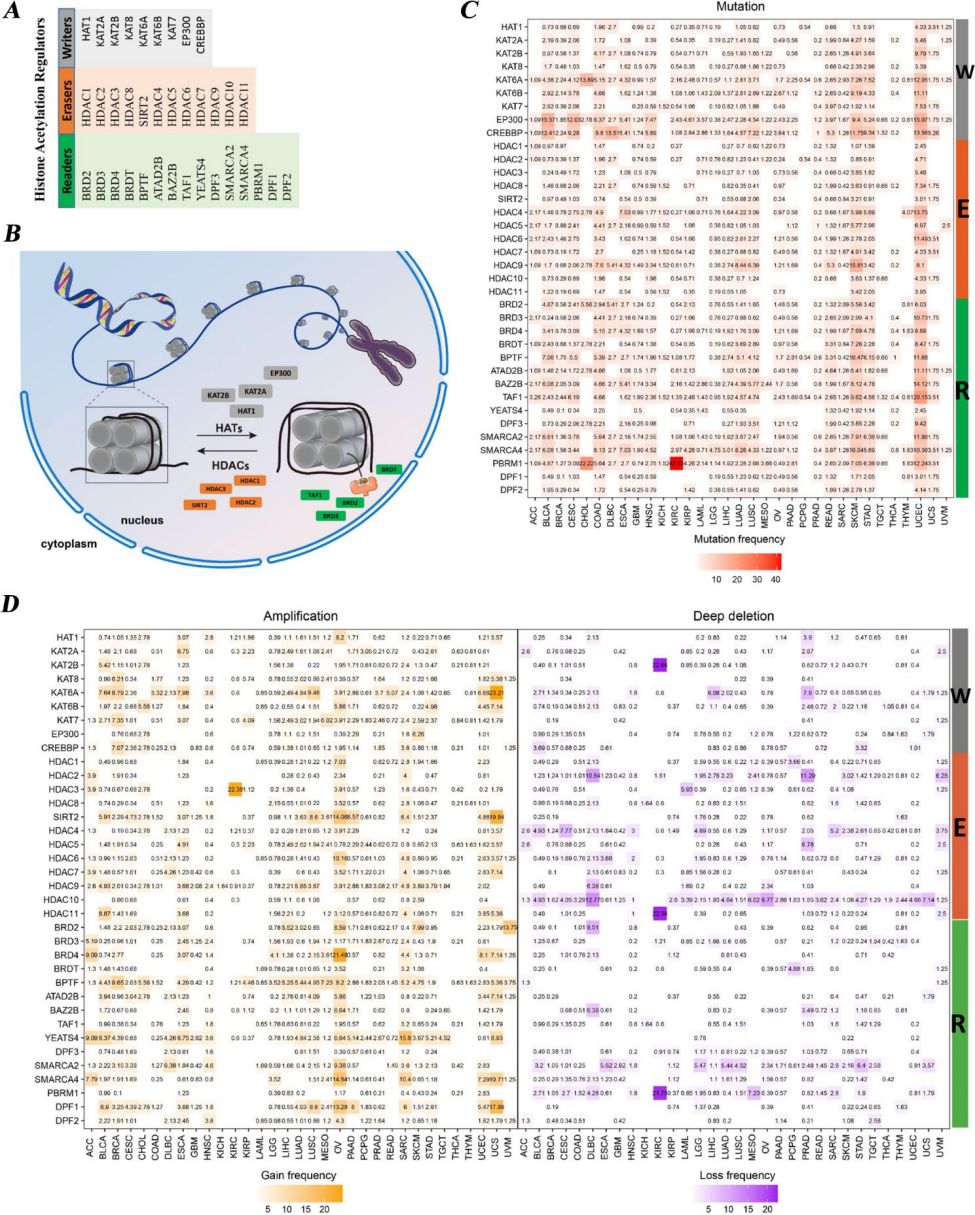
Histone acetylation (HA) regulators and their genetic alternations in human cancers. **(A)** The list of readers, erasers and writers among histone acetylation regulators. **(B)** The diagram of histone acetylation and deacetylation processes and associated regulators. **(C)** The mutation frequency of HA regulators across human cancers. **(D)** The CNV alternation frequency of HA regulators across human cancers

We identified 36 acknowledged histone acetylation (HA) regulators, including 9 (HAT1, KAT2A, KAT2B, KAT8, KAT6A, KAT6B, KAT7, EP300 and CREBBP) in “writers”, 12 (HDAC1, HDAC2, HDAC3, HDAC8, SIRT2, HDAC4, HDAC5, HDAC6, HDAC7, HDAC9, HDAC10, and HDAC11) in “erasers” and 15 (BRD2, BRD3, BRD4, BRDT, BPTF, ATAD2B, BAZ2B, TAF1, YEATS4, DPF3, SMARCA2, SMARCA4, PBRM1, DPF1, and DPF2) in “readers” (Fig. 1A, and Table S1). In Fig. 1B, we initially characterized the key players of these three classes of HA regulators. Generally, histone acetylation is a post-translational modification dynamically regulated by three categories of regulators, namely “writers”, “readers” and “erasers”, to maintain this trimmed process to functional execution. The “writers” transfer acetyl to N-terminal lysine residue of histones in the chromatin to neutralize the electrostatic attraction between DNA chain and histones, thus loose the nucleosome structure to promote chromatin accessibility and specific genes transcription, whereas the “erasers” catalyze the converse process. The “readers”, regarded as effector proteins, refer to recognized site-specific histones acetylation modifications and make it interpretable as well as to perform diverse functions.

Subsequently, by deeply exploring the patterns of genomic alterations across 33 cancer types, we found low overall levels of mutation frequencies in HA regulators, with the vast majority of HA regulators having less than 5% in the vast majority of cancer types (Fig. 1C). The mutation frequency of HA regulators varies with cancer contexts and regulators. In CHOL, KICH, MESO, PCPG, TGCT, THYM, and UVM, about three quarters of the regulators do not have any mutations. The “reader” PBRM1 has a high mutation frequency in KIRC and CHOL, at 42.42% and 22.22%, respectively, without any mutations in PCPG, THCA and UVM. We found that the overall mutation frequency of HA regulators was higher in UCEC and SKCM than in other cancers, which is consistent with a previous report [10].

We noticed that HA regulators exhibits a cancer-type-dependent pattern of CNV amplification or deep deletion (Fig. 1D). For example, in KICH, the vast majority of HA regulators did not show CNV amplification or deep deletion, but the opposite occurred in OV. Furthermore, HDAC3 had relatively high CNV amplification (22.36%) and lacked deep deletions in KIRC. However, KAT2B, HDAC11, and PBRM1 had a relatively high CNV deep deletion and lacked amplification in KIRC. The genomic alterations of HA regulators in UCEC are dominated by somatic mutations, while in OV, CNV amplification dominated them. Collectively, these results (Fig. 1B-D and Table S2) reveal a highly heterogeneous genetic alteration landscape of HA regulators across different human cancer types.

### Aberrant expression of HA regulators among cancer types

**Fig. 2:**
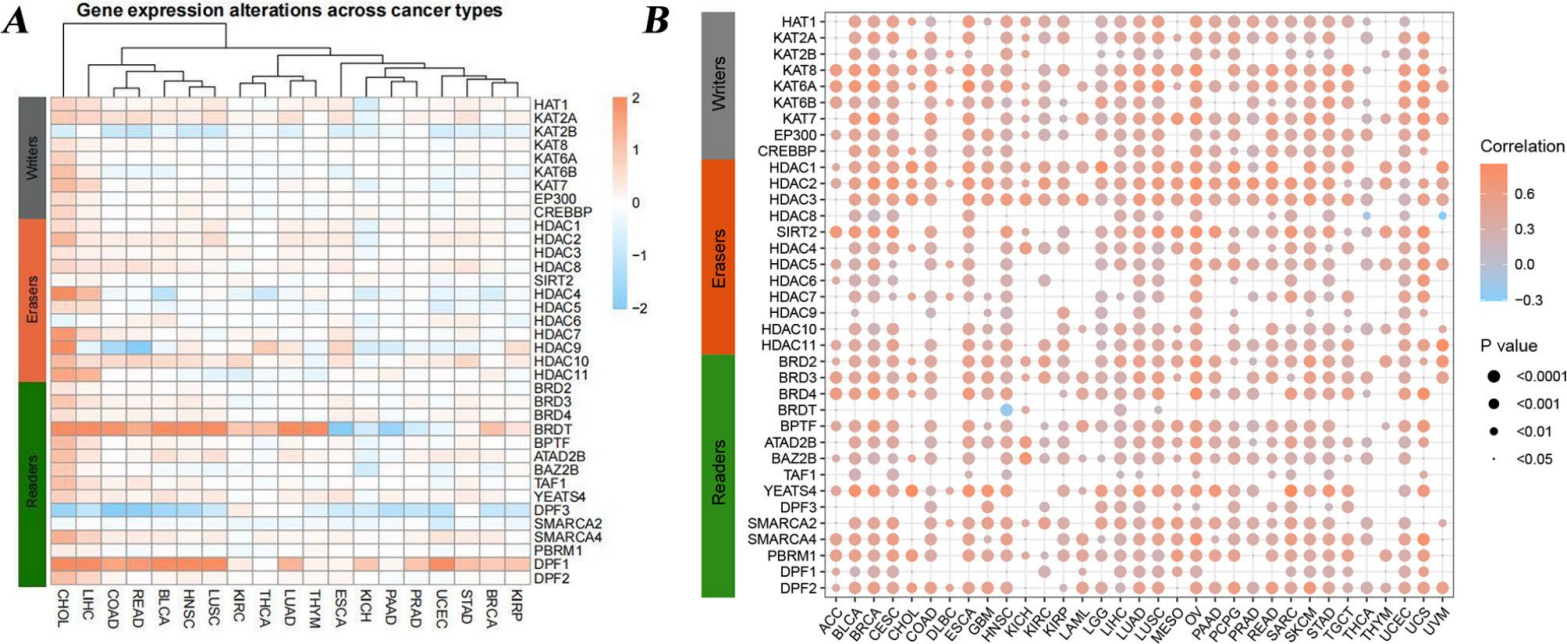
The dysregulated gene expression of HA regulators across human cancers. **(A)** The heatmap showing different expression alterations of HA regulators in human cancers. **(B)** The Pearson correlation between somatic copy number alterations and the gene expression of HA regulators

We previously investigated the genetic alteration of HA regulators in human different cancer types. To make as clear as possible the potential associations between these alterations and HA regulators expression, we therefore performed differential expression analysis to explore the expression perturbations of HA regulators in 19 cancer types (with at least three normal control samples), and Pearson correlation analysis to investigate the associations between somatic CNV and the expression of HA regulators. We found that the vast majority of HA regulators are differentially expressed in most cancer types. Several HA regulators showed consistent expression patterns in cross-cancer analyses. HDAC3, HDAC10, HDAC2, HDAC7, ATAD2B were significantly upregulated in 9, 14, 13, 9, and 10 cancer types, respectively, whereas KAT2B was downregulated in 16 cancer types (Fig. 2A and Table S3). In addition, the expression patterns of some HA regulators are cancer-type-dependent. For example, HDAC3 was upregulated in most cancer types, including LUAD (log2FC = 0.18, adj.P = 2.94E-09) and LIHC (log2FC = 0.26, adj.P = 1.36E-07), but was down-regulated in KICH (log2FC = -0.21, adj.P = 2.53E-04) and THCA (log2FC = -0.07, adj.P = 1.15E-04). This suggests that HA regulators may play different roles in different cancer types. Our results suggest that CNV alteration may be one of the major mechanisms leading to the expression perturbations in HA regulators. Compared with normal tissues, the expression of CNV-amplified HA regulators was significantly increased in tumor tissues (e.g. HDAC3 in KIRC), while the expression of CNV-deficient HA regulators was significantly decreased (e.g. KAT2B, HDAC11 and PBRM1 in KIRC). Further, we investigated the Pearson correlation between HA regulators gene expression and copy number. We found that the expression of most HA regulators was significantly positively correlated with copy number, except for BRDT in HNSC, HDAC8 in THCA and UVM (Fig. 2B and Table S4). These results suggest that the copy number of HA regulator is abnormal in most cancer types and can affect gene expression. This highlights the importance of the dysregulation of HA regulators in different cancers.

### Association between HA regulators and Hallmark pathways in Cancer

**Fig. 3:**
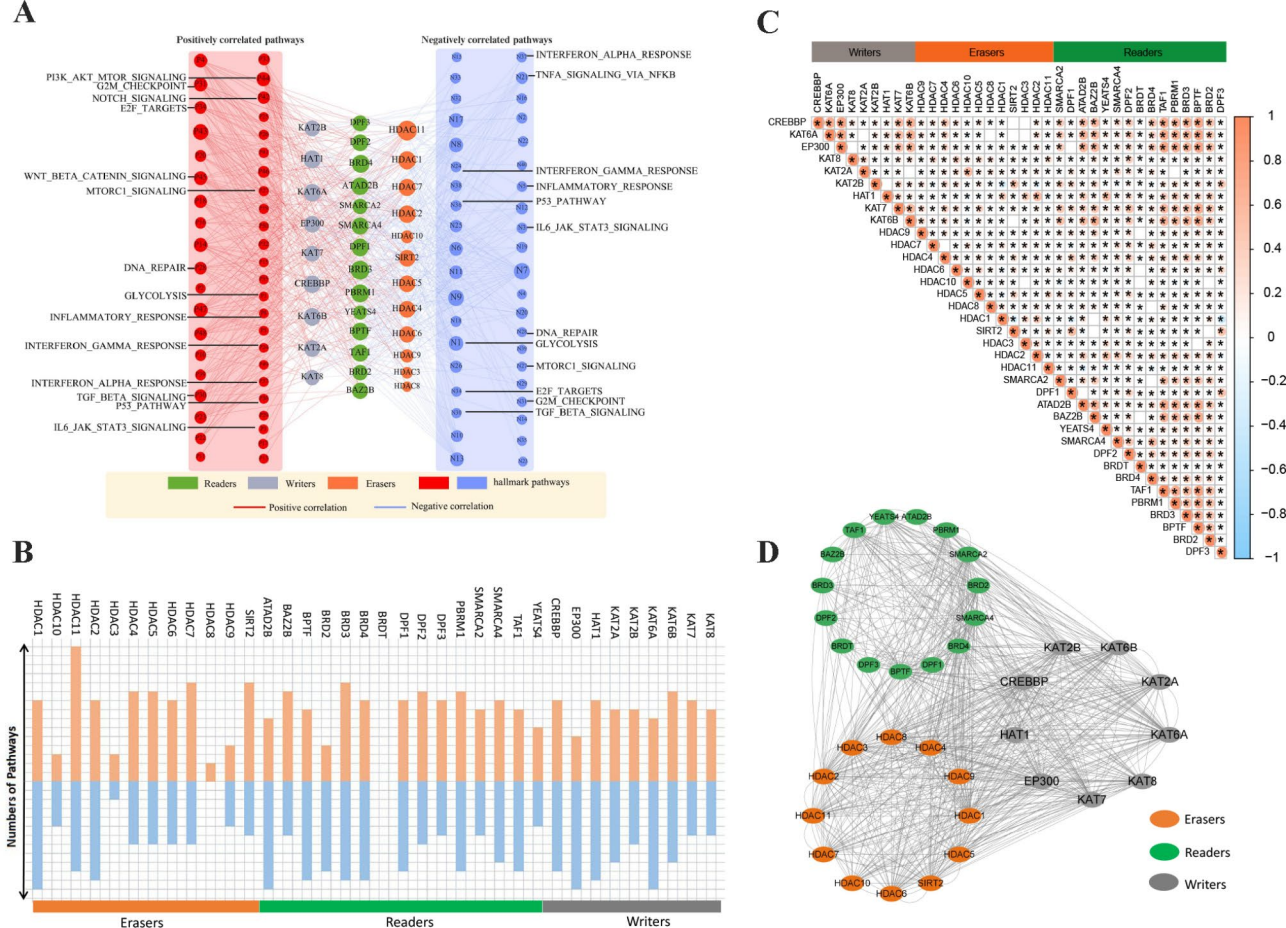
Association of the HA regulators with Hallmark oncogenic pathways. **(A)** Network diagram showing the correlation between the expression of HA regulators and the activity of cancer pathways. **(B)** The number of pathways involved by each HA regulator. **(C)** Pearson correlation among HA regulators. **(D)** The protein-protein interaction (PPI) networks among these HA regulators

To further elucidate the molecular mechanisms underlying the involvement of HA regulators in cancer, we calculated the correlation between the expression of individual HA regulators and the activity of 50 Hallmark pathways. We observed that the expression of each HA regulator was associated with the activation or inhibition of multiple pathways (Fig. 3A and Table S5). For example, HDAC3 was associated with the activity of 46 pathways, of which 23 were positively related (including E2F Targets, G2M Checkpoint, MYC Targets V1, Epithelial Mesenchymal Transition, PI3K/AKT/mTOR signaling), and 23 were negatively related (including Bile acid metabolism, Protein Secretion). Further reinforcing this correlation (|PCC|>0.2 and adj.P < 0.001), we found that HDAC11 was associated with the most pathway activity, which was associated with inhibition of 9 pathways and activation of 15 pathways (Fig. 3B and Table S5). HA regulators of the same functional category are associated with different pathway activities, and HA regulators of different functional categories (“readers”, “writers” or “erasers”) are associated with the same pathway activity, indicating that there is functional crosstalk among HA regulators, that is, HA regulators in the same functional category have different functional roles.

Previous studies [15, 16]have shown close collaboration among “readers”, “writers” and “erasers” belonging to different functional categories in cancer. Our results also preliminarily demonstrate this point. We found that not only HA regulators within the same functional class showed significantly correlated expression patterns (e.g. CREBBP-KAT6A, CREBBP-EP300), but also high correlations between different functional classes (e.g. CREBBP-PBRM1, CREBBP-BRD3, EP300-BRD3) (Fig. 3C). Furthermore, we also noted extensive interactions of these regulators in the protein-protein interaction (PPI) network (Fig. 3D). Taken together, these results suggest that cross-talk among “readers”, “writers” and “erasers” of HA plays key roles in different cancer types.

#### Clinical relevance of HA regulators

**Fig. 4:**
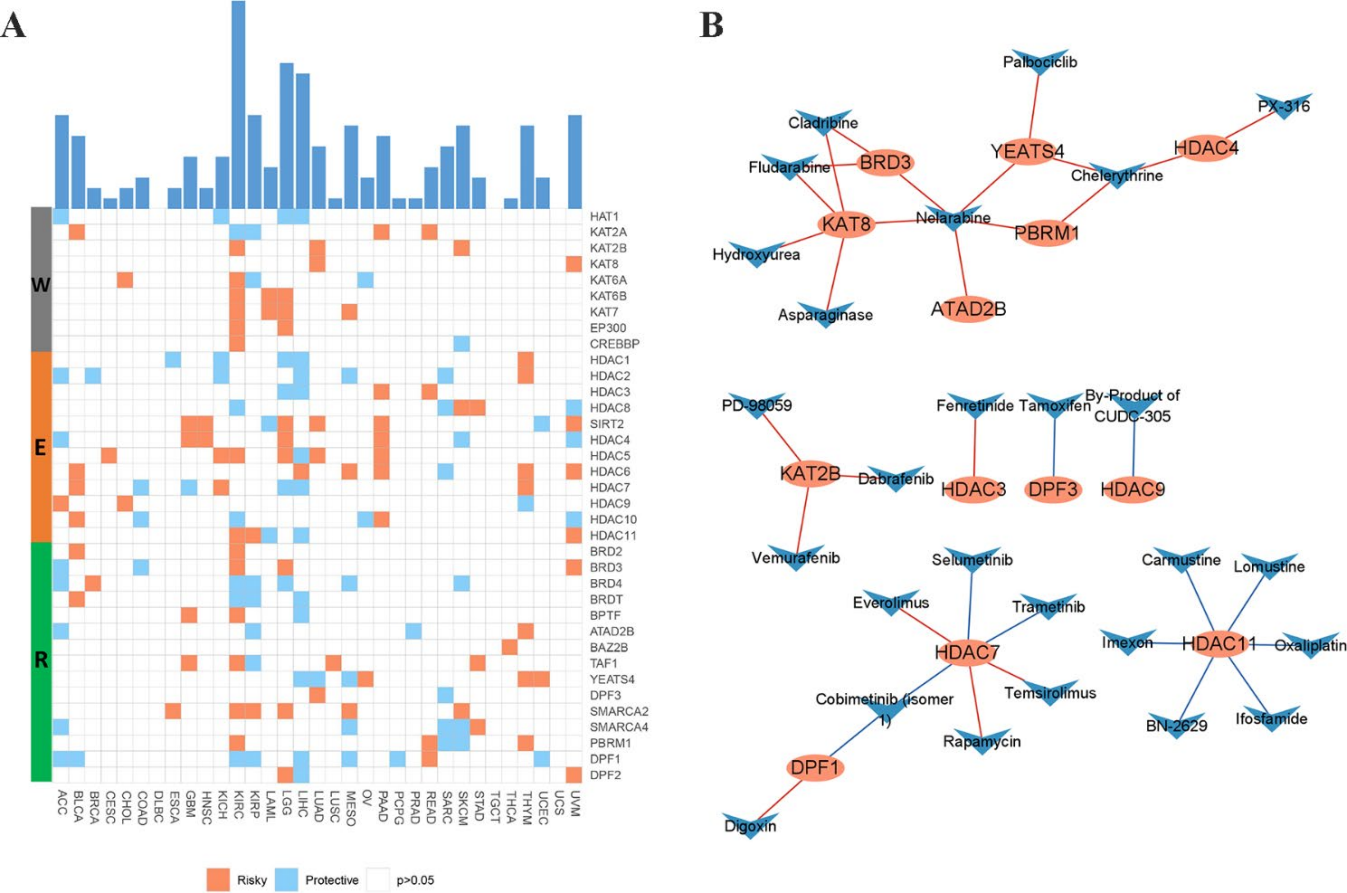
Clinical relevance of HA regulators. **(A)** Prognostic predictive value of individual HA regulators for patients with different cancer types. **(B)** The correlation between the expression of HA regulators and drug IC50. Red lines represent positive correlation and blue lines represent negative correlation

Given the highly heterogeneous genetic and expression alterations of HA regulators in cancer, it is warranted to further investigate the clinical relevance of the HA regulators. First, we investigated the relationship between HA regulators and patient survival (Fig. 4A). We found a significant cancer-specific relationship between HA regulators and patients’ prognosis. For example, HDAC3 is a prognostic unfavorable factor in PAAD and READ, but the opposite factor occurs in LIHC and LGG. Regulators in the same functional category in the same cancer type may also present radically different prognostic values. For example, HDAC2 and HDAC9, both “readers”, had opposite effects on survival in ACC patients. Subsequently, to screen for potential drugs targeting HA regulators, we downloaded drug information and RNA-seq data from the CellMiner database for drug sensitivity analysis. The results showed that HDAC11, HDAC7, BRD 3, YEATS4, and HDAC3 correspond to 6, 6, 3, 3, and 1 drugs, respectively (Fig. 4B and Table S6). For example, the expression of HDAC7 was negatively correlated with the IC50 of selmetinib and cobimetinib, suggesting that its high expression may enhance the drug sensitivity of selmetinib and cobimetinib.

**Fig. 5:**
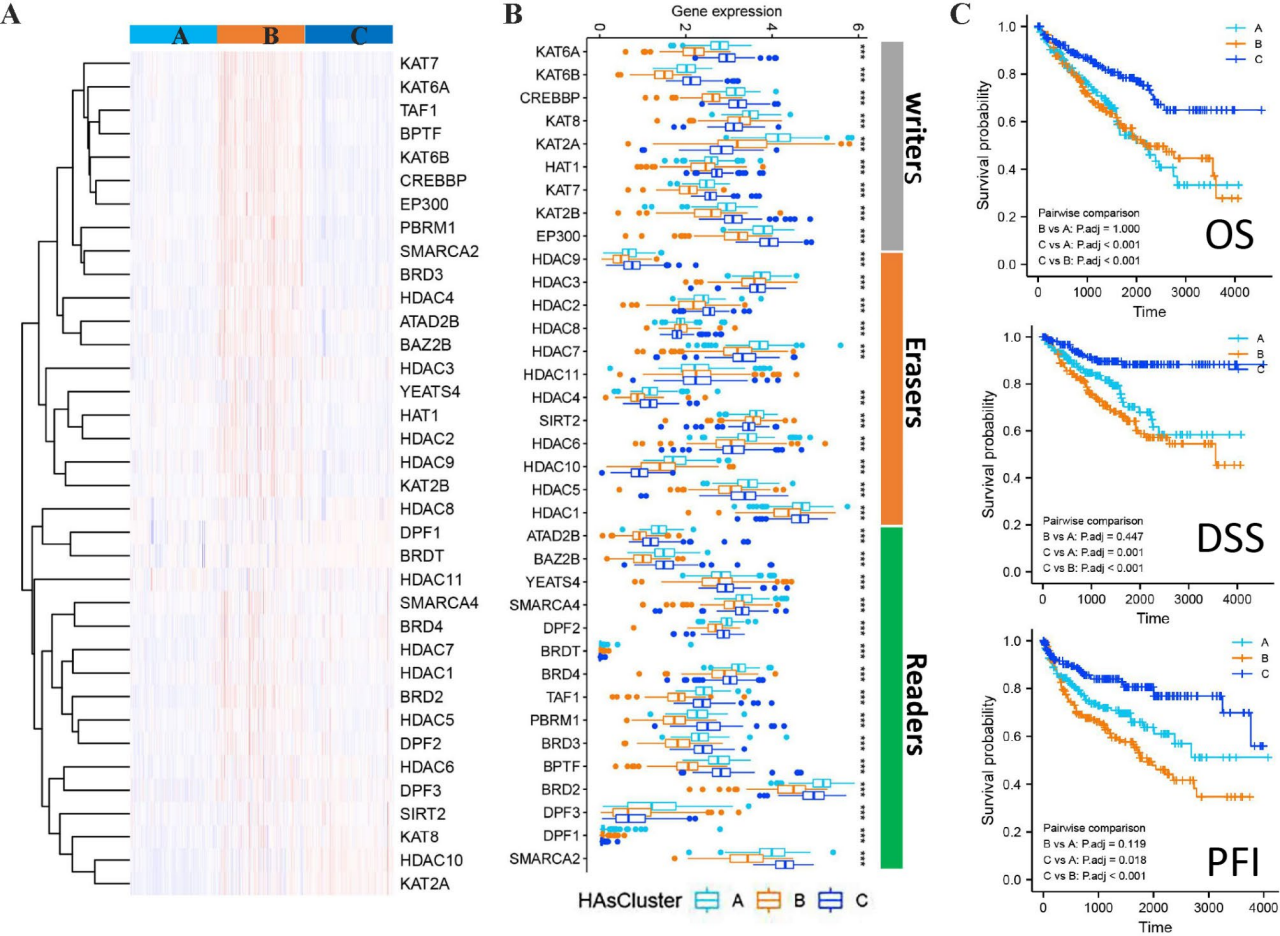
The unsupervised clustering identified three patient clinical clusters for KIRC. **(A)** The heatmap showing the clustering for KIRC patients based on the overall expression pattern of HA regulators. **(B)** Differential expression of HA regulators among the three clusters. **(C)** The Kaplan-Meier survival plots showing the survival differences among the three clusters

Among these 33 cancer types, we observed that KIRC had the largest number of prognosis-related regulators (20/36). In order to explore whether the expression of HA regulators contributes to the prognostic stratification of KIRC, we performed unsupervised clustering based on the overall expression pattern of HA regulators and finally identified three patient clinical clusters. We called them HAsCluster A, HAsCluster B, and HAsCluster C. They contained 175, 177, and 183 patients, respectively. Figure 5 A and Fig. 5B show the overall expression landscape of HA regulators among three clinical clusters in heatmap and bar-graphs, respectively. We found that the vast majority of the HA regulators had the lowest expression in HAsCluster B. We subsequently explored survival differences among the three clusters (Fig. 5C). The results showed that HAsCluster C had significantly better clinical outcomes, no matter in OS, DSS or PFI. Overall, our findings demonstrate the great potential of HA regulators both in the generation of novel therapeutic strategies and in the prognostic stratification of specific cancer types.

### Validation of the oncogenic role of HDAC3 in LUAD

**Fig. 6:**
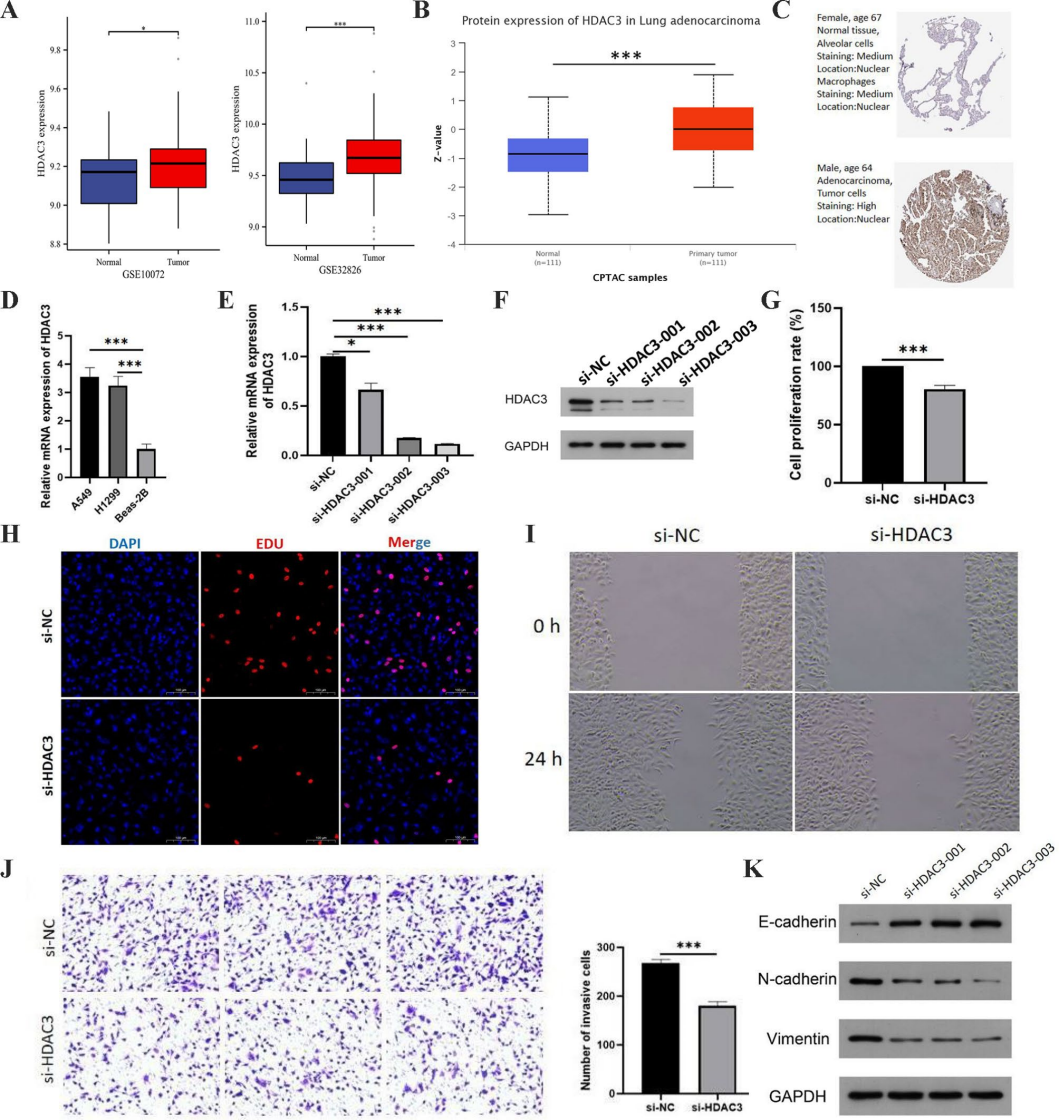
Validation of the oncogenic role of HDAC3 in LUAD. **(A)** Differential mRNA expression of HDAC3 between LUAD tumor and normal tissues: results from two GEO chips (GSE10072 and GSE32826). **(B)** Differential protein expression of HDAC3 between LUAD tumor and normal tissues: results from CPTAC samples. **(C)** Differential protein expression of HDAC3 between LUAD tumor and normal tissues: results from HPA. **(D)** Differential expression of HDAC3 between LUAD cells (A549 and H1299) and normal bronchial epithelial cells (Beas-2B). **(E)** qPCR validation of RNA interference effects. **(F)** Western blot validation of RNA interference effects. **(G)** CCK-8 assay reveals the cell proliferation rate of si-HDAC3 group and si-NC group. **(H)** Edu assay reveals the cell proliferation of si-HDAC3 group and si-NC group. **(I)** Woundhealing assay reaveals the cell migration of si-HDAC3 group and si-NC group. **(J)** Transwell assay reveals the cell invasion of si-HDAC3 group and si-NC group. **(K)** Western blot reveals the expression levels of epithelial mesenchymal transition related proteins (E-cadherin, N-cadherin, Vimentin) in different groups of cells

Histone deacetylases (HDACs) are cellular enzymes that act a crucial role in epigenetic regulation of gene expression by remodeling chromatin [17, 18]. HDAC3, an important member of class I HDACs functioning in many key cellular processes, has recently been found to be overexpressed in multiple malignancies and is considered to be one of the most frequently upregulated genes in malignancies[19, 20]. Considering that the critical functions of HDAC3 in human cancers including LUAD are still not fully interpreted and the important findings were observed in this study, we further revealed the important role of HDAC3 in LUAD through cell function experiments. HDAC3 was highly expressed in multiple cancer types, including LUAD. We also observed a similar situation in two LUAD GEO chip datasets (GSE10072 and GSE32826) (Fig. 6A). This expression pattern was also validated at the protein level (Fig. 6B-C). Subsequently, we also explored at the cellular level and found that HDAC3 was more highly expressed in two LUAD cell lines (A549 and H1299) compared to normal bronchial epithelial cells (Beas-2B) (Fig. 6D). We interfered the expression of HDAC3 with siRNA to further investigate the effect of HDAC3 on the malignant biological behavior of cells. We found that RNA interference significantly reduced HDAC3 expression (Fig. 6E-F). The absorbance at 450 nm represents the number of cells, reflecting the capacity of cell proliferation. According to the results of CCK-8 assay, the cell proliferation rate of si-HDAC3 group was significantly lower than that of si-NC group (Fig. 6G). Also in the EdU assay, the number of EdU-positive cells in the si-HDAC3 group was significantly lower than that in the si-NC group after RNA interference treatment of A549 for 24 h (Fig. 6H). In the subsequent wound healing assay, we noted that after RNA interference treatment of A549 for 24 h, the scratch area of si-HDAC3 group was significantly higher than that of si-NC group (Fig. 6I), indicating that knockdown of HDAC3 could inhibit cell migration of A549. According to the results of Transwell invasion assay, we also found that the invasion ability of A549 was significantly decreased after RNA interference treatment (Fig. 6J). In the investigation results mentioned above, in addition to the observation of high HDAC3 expression in LUAD, we also found that HDAC3 was associated with the activation or inhibition of multiple Hallmark pathways. Our bioinformatic results initially suggested that HDAC3 might participate in the epithelial mesenchymal transition process, and this conclusion was also confirmed by in in vitro experiments. In western blot, 24 h after RNA interference treatment of A549, it was observed that the expression levels of E-cadherin in si-HDAC3 group were significantly higher than those in si-NC group, while the expression levels of N-cadherin and Vimentin were significantly lower than those in si-NC group (Fig. 6K). This suggests that knockdown of HDAC3 inhibits epithelial mesenchymal transformation in human LUAD cells. Collectively, these results suggested that HDAC3 played an oncogenic role in LUAD.

## Discussion

HA is a post-translational modification that controls the transcript level of genes and plays a key role in the structural modification of chromosomes and the regulation of gene expression [21, 22]. Increasing evidence suggests that HA is closely related to tumor occurrence and development [23, 24]. For example, histone deacetylases can affect gene expression by direct association with transcription factors, which are deacetylated in malignancy [25]. Bianco-Miotto T et al. found that in prostate cancer, specific histone modifications could predict the progression of cancer [26]. Similarly, in breast cancer, Elsheikh S et al. also observed the close relationship between global histone modification and tumor phenotype and patient prognosis [27]. Molecular drugs targeting HA regulators (e.g. HDAC inhibitors) have been shown to have effective anti-tumor effects in haematological and solid malignancies [28, 29]. With the deepening of research, HA is also considered to be closely related to the tumor immune microenvironment [30]. HDAC inhibitors can reshape the tumor microenvironment through a variety of mechanisms, thereby enhancing the ability of immune surveillance and killing tumor cells. These studies have highlighted the importance of HA in tumor development as well as in anti-cancer drug development. However, existing analyses are limited to a single HA regulator, and the role of HA modification is characterized by highly integrated interactions among many regulators. Therefore, a comprehensive understanding of the regulatory network of HA regulators and their important roles in cancer will facilitate the development of anti-cancer therapeutic strategies.

In this study, we retrospectively investigated genomic data from more than 10,000 tumor samples from TCGA project. We found that the 36 HA regulators had low levels of overall mutation frequency in human cancers, with mutation frequencies varying with cancer context and regulators. Furthermore, we also observed a higher overall mutation frequency of HA regulators in UCEC and SKCM than in other cancers, which was consistent with a previous report [10]. We investigated the correlation between gene expression of the HA regulators and copy number, and found that the expression levels of almost all the regulators were positively correlated with copy number. Compared with normal tissues, the expression of CNV-amplified HA regulators was significantly increased in tumor tissues, while the expression of CNV-deficient HA regulators was significantly decreased, suggesting that the CNV alteration may be one of the main mechanisms leading to expression perturbation of HA regulators.

In the analysis of HA regulator related oncogenic pathways, we observed that the expression of each HA regulator was associated with the activation or inhibition of multiple pathways. Among them, HDAC3 was shown to be associated with the activity of several pathways, including E2F target, G2M checkpoint, MYC target V1, epithelial mesenchymal transition, and PI3K/AKT/mTOR signaling pathway and so on. HDAC3 has been shown to regulate the biological activities of colon cancer cells, including proliferation, differentiation and apoptosis [31, 32]. Moreover, HDAC3 was also confirmed to be overexpressed in triple-negative breast cancer [33], histone deacetylase inhibitors (I-7ab) specifically reduced HDAC3 expression and promoted acetylation of p53 to induce expression of p21, resulting in cell cycle arrest in G1 phase [34]. HDAC3 was reported to be an important player in the development of acute promyelocytic leukemia, and knockdown of HDAC3 can inhibit the PI3k/Akt-mediated signaling pathways and the induction of Caspase activity, thus leading to cell death and apoptosis [35, 36]. HDAC3 has also been found to be involved in the development of many other malignancies, such as melanoma[37], gastric cancer [38], ovarian cancer[39], and children glioma [40]. The important role of HDAC3 in LUAD is still undercharacterized. This study clarified its oncogenic role in LUAD based on bioinformatics and in vitro experiments. In A549, knockdown of HDAC3 could significantly inhibit malignant biological behaviors such as cell proliferation, migration, and invasion, indicating an oncogenic function of HDAC3 in LUAD, which was consistent with it in other cancer types. Epithelial mesenchymal transition is an important biological process for epithelial cell-derived malignant tumor cells to acquire the ability of migration and invasion. In fact, the relationship between HDAC3 and epithelial mesenchymal transition has been reported in malignant tumors and other diseases [41, 42]. For example, targeting HDAC3 can block epithelial mesenchymal transition plasticity in gastric cancer[43], HDAC3 can enhance the migration and invasion properties of fibroblasts by positively affecting the epithelial mesenchymal transition process [44]. In this study, we found that the knockdown of HDAC3 could significantly inhibit the epithelial mesenchymal transition of A549, further revealing an important link between HDAC3 and epithelial mesenchymal transition. Although the oncogenic role of HDAC3 in LUAD has been identified, the specific mechanism remains to be explored. Considering that HDAC3 is a histone deacetylase encoding gene, future exploration of its carcinogenic mechanism can focus on the bridge relationship between the deacetylation modification of downstream regulatory factors by HDAC3 and epithelial mesenchymal transformation.

This study identified genomic alterations and expression perturbations of HA regulators in a variety of cancers, and we are therefore interested in further investigating their clinical translational value. We found a significant cancer-specific relationship between HA regulators and patient outcomes. Of these 33 cancer types, KIRC had the most prognosis-related regulators. Thus, we further explored the significance of the expression of HA regulators in the prognostic stratification of KIRC. We found that KIRC patients could be classified into three clinical clusters with different prognostic characteristics (HAsCluster A, HAsCluster B, and HAsCluster C). This suggested that the transcription profile signature based on HA regulators had prognostic stratification significance. The vast majority of HA regulators were least expressed in HAsCluster B, suggesting that the activity of HA might be relatively low in HAsCluster B. Surprisingly, HAsCluster B had significantly better clinical outcomes, either in terms of OS, DSS, or PFI. This suggested that there might be no one-to-one correspondence between HA and patient outcomes. The development of genomics drives the discovery of tumor-related genes, which will greatly promote the development of anti-cancer drugs. We combined the CellMiner database to explore potential targeted drugs for HA regulators. Using stringent screening criteria, we identified 36 gene-drug pairs involving 13 genes and 27 drugs. These agents was in clinical trials or approved by the FDA, suggesting that they might be potential anti-cancer agents targeting HA regulators, which would provide reference for the development of anti-cancer clinical drugs for follow-up studies. Selumetinib was one of the drugs identified by our CellMiner-based drug sensitivity analysis. Selumetinib was an inhibitor of mitogen-activated protein kinase 1 and 2 (MEK1/2), which were an upstream regulator of the ERK pathway and were frequently activated in a variety of cancers[45–47]. Our results revealed correlation of selumetinib and HDAC7, which has been rarely been highlighted in previous studies. This result may provide some direction for future clinical anti-cancer drug development, but still needs further experimental exploration. Taken all together, our results demonstrate the great potential of HA regulators in developing novel therapeutic strategies and in prognostic stratification in specific cancer types.

Overall, this study presents a comprehensive analysis of multiple levels of data on HA regulators in more than 10,000 patients, including somatic mutations, copy number variations, mRNA expression, prognostic value, potential target drugs, etc. This study systematically revealed the molecular features and clinical significance of HA regulators in human cancers, but several key questions still need to be addressed. First, the description of the molecular characteristics of HA regulators in this study is mainly based on the TCGA platform, and there is still a lack of further verification of other platforms and appropriate molecular biology experiments. Although we identified the oncogenic role of HDAC3 in LUAD by cell function experiments, the specific molecular mechanisms remain unelucidated. Secondly, this study only preliminarily identified 36 gene-drug pairs, and a total of 27 drugs may have anti-HA regulator effects, but whether they have anti-cancer activity remains to be investigated. In conclusion, our study for the first time revealed the important role of HA regulators in human cancer, which provides a rich resource for understanding the biology of HA regulators and a new perspective for developing cancer therapeutic strategies based on HA modification.

### Electronic supplementary material

Below is the link to the electronic supplementary material.

Supplementary Material 1

Supplementary Material 2

Supplementary Material 3

Supplementary Material 4

Supplementary Material 5

Supplementary Material 6

## Data Availability

The datasets generated and analysed during the current study are available in the TCGA GDC repository (https://portal.gdc.cancer.gov), GEO repository (GSE10072 and GSE32863), (https://www.ncbi.nlm.nih.gov/geo/), and so on.

## Abbreviations

HA: Histone acetylation
TGGA: The Cancer Genome Atlas
ACC: Adrenocortical Carcinoma
BLCA: Bladder Urothelial Carcinoma
BRCA: Breast Cancer
CESC: Cervical Aquamous Cell Carcinoma And Endocervical Adenocarcinoma
CHOL: Cholangiocarcinoma
COAD: Colon Adenocarcinoma
DLBC: Lymphageal neoplasm diffuse large b-cell lymphoma
ESCA: Esophageal Caecinoma
GBM: Glioblastoma Multiforme
HNSC: Head And Neck Squamous Carcinoma
READ: Rectum Adenocarcinoma
KICH: Kidney Chromophobe
KIRC: Kidney renal clear cell carcinoma
KIRP: Kidney Renal Clearn Cell Carcinoma
LAML: Acute Myeloid Leukemia
LGG: Brain Lower Grade Glioma
LIHC: Liver Hepatocellular Carcinoma
LUAD: Lung adenocarcinoma
LUSC: Lung Squamous cell carcinoma
MESO: Mesothelioma
OV: Ovarian serous cystadenocarcinoma
PADD: Pancereatic Adenocarcinoma
PCPG: Pheochromocytoma And Paraganglima
PRAD: Prostate Adenocarcinoma
SARC: Sarcoma
SKCM: Skin Cutaneous Melanoma
STAD: Stomach Adenocarcinoma
TGCT: Testicular Germ Cell Tumors
THCA: Thyroid Carcinoma
THYM: Thymoma
UCEC: Uterine Corpus Endometrial Carcinoma
UCS: Uterine Carcinosarcoma
UVM: Uveal Melanoma
GSEA: Gene-set enrichment analysis
CNV: Copy number variation
GSVA: Gene set variation analysis
SCNA: Somatic copy number alterations
